# Epidemiological trends and age-period-cohort effects on subarachnoid hemorrhage burden across the BRICS-plus from 1992 to 2021

**DOI:** 10.3389/fmed.2025.1582357

**Published:** 2025-07-03

**Authors:** Liang Guo, Dan Song, Li Chen, Ying Huang

**Affiliations:** ^1^Dermatology Hospital of Jiangxi Province, Nanchang, Jiangxi, China; ^2^Jiangxi Provincial Clinical Research Center for Skin Diseases, Nanchang, Jiangxi, China; ^3^Candidate Branch of National Clinical Research Center for Skin Diseases, Nanchang, Jiangxi, China; ^4^JXHC Key Laboratory of Skin Infection and Immunity, Nanchang, Jiangxi, China; ^5^The Affiliated Dermatology Hospital of Nanchang University, Nanchang, Jiangxi, China

**Keywords:** subarachnoid hemorrhage, BRICS-plus, incidence, age-period-cohort model, public health, trend

## Abstract

**Background:**

Subarachnoid hemorrhage (SAH) is a major global health concern associated with disproportionately high morbidity and mortality. The BRICS-plus nations (Brazil, Russian Federation, India, China, South Africa, and six other new members), account for a substantial proportion of the global population while being confronted with distinct public health challenges. This study aims to examine epidemiological trends and regional variations in SAH burden across BRICS-plus nations through comprehensive and timely analysis.

**Methods:**

Data on the number, all-age rate, age-standardized rate, and relative change in SAH incidence from 1992 to 2021 across eleven BRICS-plus members were sourced from the Global Burden of Disease Study (GBD) 2021. Associations between the incidence rate and the Socio-demographic Index (SDI) were assessed through Pearson correlation analyses. Furthermore, age-period-cohort modeling was utilized to quantify net drift, local drift, age, period, and cohort effects over the past three decades.

**Results:**

Except for China, SAH cases were observed to have significantly increased in the other ten BRICS members from 1992 to 2021. All BRICS-plus countries exhibited a declining trend in the age-standardized incidence rate over the study period. Indonesia reported the highest age-standardized incidence rate (10.94 per 100,000 population) in 2021, while China displayed the most significant decrease, at 59.36%. The annual net drift in the SAH incidence rate ranged from −3.36%% for China to −0.50% for the Russian Federation among the eleven countries. A significant negative correlation was observed between the incidence rate of SAH and SDI values. Nations displayed similar age-effect patterns characterized by initial declines followed by subsequent increases with advancing age, along with distinct period and cohort effects that may reflect variations in control measures and temporal burden patterns.

**Conclusion:**

Our study demonstrates the overall decline in age standardized incidence rate of SAH, while highlighting the persistent health inequalities among eleven countries potentially attributable to socioeconomic disparities. Furthermore, the findings underscore the imperative for tailored interventions across age, period, and cohort dimensions to mitigate SAH-specific challenges in nations undergoing rapid development.

## 1 Introduction

Subarachnoid hemorrhage (SAH), a life-threatening subtype of hemorrhagic stroke, occurs when there is bleeding into the subarachnoid space—the cerebrospinal fluid-filled compartment between the arachnoid mater and pia mater, which envelops the brain and spinal cord (1). Although SAH accounts for approximately 5–10% of all strokes, it is associated with disproportionately high morbidity and mortality, with half of all cases resulting in death within the first two weeks (2). Epidemiologically, SAH primarily affects adults aged 40–60 years, a demographic peak coinciding with the peak of workforce productivity, thereby imposing significant societal and economic burdens (3, 4). The abrupt onset of SAH, accompanied by frequent complications such as rebleeding, cerebral vasospasm, and delayed cerebral ischemia, underscores its status as a chronic public health issue (5). Furthermore, the anticipated rise in SAH burden due to global population aging necessitates urgent multidisciplinary collaboration to improve prevention and management strategies.

The term BRICS initially encompassed Brazil, Russian Federation, India, China, and South Africa, representing a group of fast-growing economies with emerging global influence in trade, infrastructure, and public health indices (6). However, the geopolitical landscape has recently evolved significantly: starting in January 2024 and January 2025, countries such as Saudi Arabia, Egypt, the United Arab Emirates, Iran, Ethiopia, and Indonesia will formally join this alliance, expanding its representation to 11 nations (BRICS-plus). Collectively, these nations are characterized by significant population disparities, varying levels of economic development, diverse healthcare infrastructures, and increasing attention toward their growing share in the global disease burden (7). With SAH contributing to notable population-wide mortality and disability in both high- and low-resource settings, characterizing its burden in eleven BRICS countries is both critically necessary and timely. Understanding SAH’s disease trajectory and discrepancies across these nations will provide essential insights into the broader health implications faced by this emerging league of economies.

Previous investigations derived predominantly from the Global Burden of Disease (GBD) 2019 have attempted to map the complex regional heterogeneities associated with SAH-related disease burden (8, 9). These findings make it apparent that evidence-based, region-specific health policy decisions are essential, particularly in low- and middle-income regions. While descriptive epidemiology provides foundational insights through the characterization of disease burden, it fails to capture the temporal dynamics and nuanced interplays of age, period, and cohort effects on incidence trends. The introduction of Age-Period-Cohort (APC) modeling presents a novel approach in this realm, offering a dynamic framework to dissect these interrelations and refine epidemiological investigations for conditions like SAH more adequately (10). The recent update from the GBD 2021 database provides an opportune dataset to further probe this refined analysis (1). This comprehensive resource enables the assessment of both temporal trends and spatial heterogeneities of SAH incidence across the expanded BRICS countries consortium. Specifically, integrating APC models with the GBD 2021 can derive critical evidence pertinent to monitoring epidemiological shifts within specific national contexts. This subsequently aids in shaping effective public health interventions aimed at enhancing healthcare outcomes related to SAH.

This study evaluates the incidence of SAH from 1992 to 2021 in BRICS-plus nations, utilizing the latest GBD 2021 estimates and an APC model-based approach. Through systematic analysis of temporal trends and generation-specific variations, this research seeks to unravel the epidemiological dynamics of SAH within this unique socioeconomic framework, offering critical insights to inform the development of targeted healthcare strategies.

## 2 Methods

### 2.1 Data sources

This study utilized data from the GBD 2021 public dataset, accessible through the Global Health Data Exchange GBD Results Tool.^1^ The GBD 2021 dataset offers a comprehensive analysis of the disease burden for 371 conditions across 204 countries and territories worldwide, spanning from 1992 to 2021 (1, 11, 12). This dataset represents an extensive resource, providing detailed insights into disease burden, associated risks, mortality rates, and disability metrics, serving as a vital tool for understanding global health challenges. Notably, the GBD 2021 dataset introduced several significant enhancements, including the addition of 19,189 new Disability-Adjusted Life Years (DALYs) data sources, the inclusion of data for 12 previously unmeasured health conditions, and advanced methodological frameworks. Furthermore, the dataset incorporated the influence of the COVID-19 pandemic on the global burden of disease, offering a more nuanced perspective on contemporary health issues.

SAH cases in GBD 2021 are classified using the International Classification of Diseases, 9th edition (ICD-9 codes: 430–430.9), and 10th edition (ICD-10 codes: I60-I60.9, I62.0, I67.0-I67.1, I69.0) (1). The study analyzed global and regional incidence data for SAH, including age-standardized rates, across eleven BRICS-plus countries. The incidence was calculated as the number of new cases reported annually divided by the mid-year population size, with data spanning age groups from 0 to 94 years and the time period from 1992 to 2021. The statistical modeling process for GBD 2021 involved replicating the sample 1,000 times to estimate 95% uncertainty intervals (UIs), with confidence bounds derived from the 2.5th and 97.5th percentiles of the uncertainty distribution. Additionally, the Socio-demographic Index (SDI) for BRICS-plus countries was updated in this edition. The methodology and modeling approach for GBD 2021 have been documented in prior publications (1, 11, 12). All data were anonymized and made publicly accessible, and the study protocol, which included a waiver for informed consent, was approved by the University of Washington Institutional Review Board.

### 2.2 Data analysis

#### 2.2.1 Analysis of overall temporal trends in SAH incidence

To assess temporal trends in incidence from 1992 to 2021, we analyzed the number of cases, all-age and age-standardized incidence rates, as well as their calculated relative percentage changes. The relationship between the Socio-demographic Index (SDI) values of eleven countries and age-standardized incidence rate was analyzed using the Pearson correlation coefficient. Finally, we examined the age distribution of SAH burden, categorizing cases into five age strata (0–4, 5–19, 20–39, 40–64, and 65–94 years), and calculated the proportion of cases in each stratum.

#### 2.2.2 Age period cohort modeling analysis

The APC model, a statistical tool grounded in the Poisson distribution, is employed to extract and uncover insights into disease trends (13, 14). In this study, the APC model was applied to disaggregate the incidence of SAH into three principal dimensions: age, period, and birth cohort, followed by an analysis of their respective influences on SAH incidence. Within this model, age effects indicate the varying risks of the outcome for different age groups. Period effects capture temporal changes that impact all age groups simultaneously, while cohort effects demonstrate variations in outcomes among individuals born in the same year. The mathematical formulation of the APC model is as follows:


$$g(Y_{j}/\mu )=\log(\lambda_{j})=u+\alpha \;{\mathrm{age}}_{j}+\beta \;{\text{period}}_{j}+\gamma \;{\text{cohort}}_{j}$$

where $\lambda_{j}$ represents the response variable of the net effect on SAH incidence rate for group j ; $Y_{j}$ and μ represent the number of incidence and the population at risk, respectively. α , β , and γ represent the coefficients of age, period, and birth cohort of the APC model, respectively. u represents the intercept of the model.

The 2021 GBD incidence estimates for SAH and corresponding population data from each country were utilized as inputs for the APC model, which incorporated the intrinsic estimator (IE) method to address parameter indeterminacy stemming from age, period, and cohort effects (15). Methodological details are extensively documented in prior literature (16). To ensure accurate model performance, the population aged 0–94 years was partitioned into 19 age groups, each spanning 5-year intervals (0–4, 5–9, …, 90–94). To mitigate the impact of transient fluctuations such as the COVID-19 pandemic, data were collected at the midpoint of six time points (1994, 1999, …, 2019) instead of relying on 5-year averages. This approach facilitated a unified framework for data assimilation, ensuring accurate representation of distinct temporal periods. The resulting input data comprised 19 age groups and 21 cohorts, defined by birth intervals ranging from 1900 to 1904 (median 1902) to 2015–2019 (median 2017).

In this study, we focused on estimating several key functions (17): (1) net drift, representing the overall annual percentage change in disease occurrence trends over time; (2) local drifts, reflecting annual percentage changes in disease occurrence by specific time periods and birth cohorts for each age group; (3) the longitudinal age curve, which shows fitted age-specific incidence rates adjusted for temporal variations within the reference cohort; and (4) the period (or cohort) rate ratio (RR), which compares age-specific incidence rates in each period (or cohort) relative to a reference period (or cohort). The APC analysis was conducted using the National Cancer Institute’s age-period-cohort web-based tool at https://analysistools.cancer.gov/apc/ (18). Subsequent data visualization and statistical analysis were performed using R software (version 4.2.3). Statistical significance for the parameters was assessed using the Wald χ^2^ test, with all tests conducted as two-tailed analyses.

## 3 Results

### 3.1 Incidence of SAH trends from 1992 to 2021

**Figure 1:**
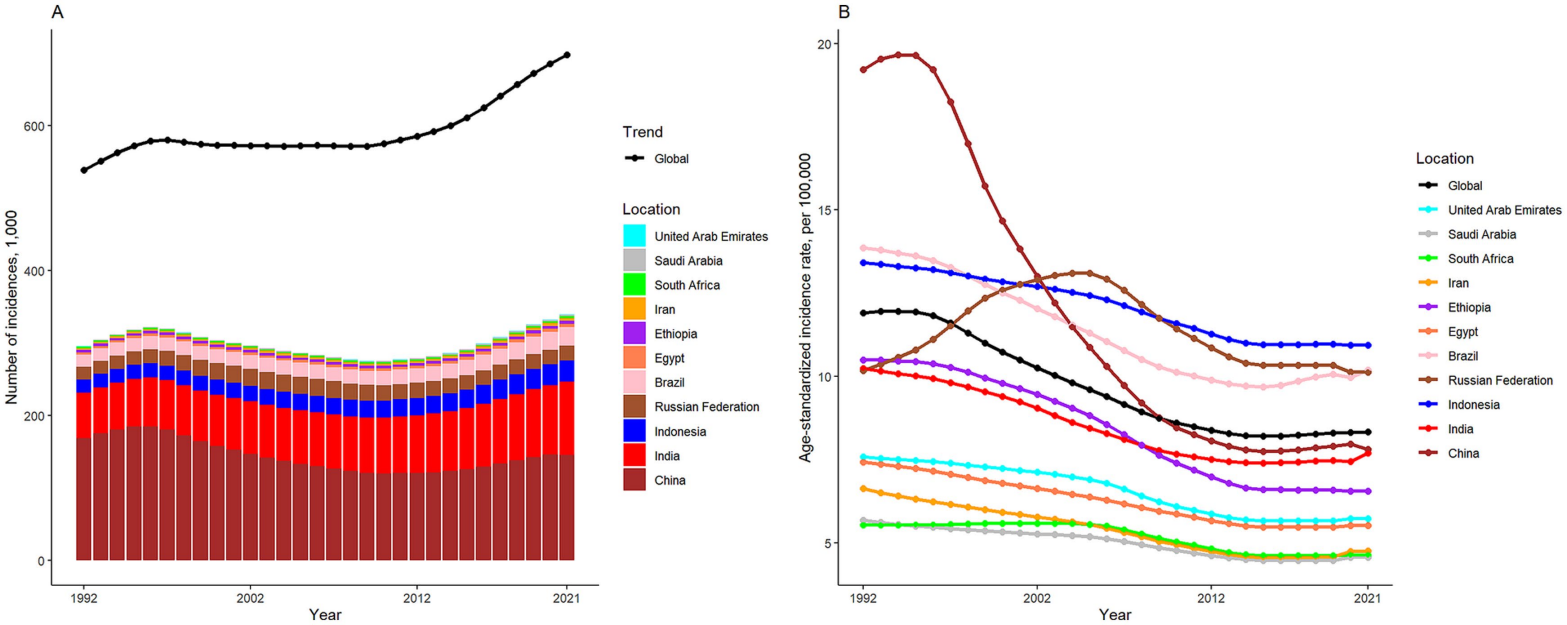
The numbers **(A)** and age-standardized rate **(B)** of subarachnoid hemorrhage incidence in global and BRICS-plus from 1992 to 2021.

Table 1 summarizes the population, total incidences, all-age incidence rate, age-standardized incidence rate, and net drift of incidence for the world and BRICS-plus countries. Over the past thirty years, there has been a significant increase in the number of SAH cases, rising from 538,452 (95% UI: 469459 to 618,203) in 1992 to 697,487 (95% UI: 614335 to 795,786) in 2021, representing a 29.54% increase (Table 1; Figure 1). Concurrently, the age-standardized incidence rate declined from 11.90 (95% UI: 10.44 to 13.69) per 100,000 population in 1992 to 8.33 (95% UI: 7.34 to 9.48) per 100,000 population in 2021, which represents a reduction of 30.05% (Table 1; Figure 1). According to the APC model, the net drift in global SAH incidence rate was −1.48% (95% confidence interval [CI]: −1.54 to −1.42) from 1992 to 2021 (Table 1).

**Table 1:** Trends in intracerebral hemorrhage incidence across the eleven BRICS countries, 1992–2021.

Characteristic	Global		Brazil		Russian Federation		India		China		South Africa		Saudi Arabia		Egypt		United Arab Emirates		Iran		Ethiopia	
	1992	2021	1992	2021	1992	2021	1992	2021	1992	2021	1992	2021	1992	2021	1992	2021	1992	2021	1992	2021	1992	2021
Population																						
Number, n x 1,000,000^*^	5,497 (5,379, 5,624)	7,891 (7,668, 8,131)	154 (143, 166)	220 (188, 251)	152 (138, 166)	145 (125, 164)	888 (823, 960)	1,415 (1,240, 1,602)	1,213 (1,117, 1,309)	1,423 (1,319, 1,530)	39 (35, 42)	57 (50, 64)	17 (16, 18)	38 (33, 43)	58 (52, 63)	106 (96, 116)	2 (2, 2)	10 (8, 11)	60 (55, 66)	85 (77, 94)	55 (50, 59)	109 (92, 125)
Percentage of global, %	100.00	100.00	2.80	2.79	2.77	1.83	16.20	17.93	22.07	18.03	0.71	0.72	0.31	0.48	1.06	1.34	0.04	0.13	1.09	1.08	1.00	1.38
Incidence																						
Number, n x 1,000^*^	64552.59 (60600.74, 68787.34)	79457.43 (72748.91, 85480.16)	1352.20 (1319.41, 1385.96)	1135.06 (1081.88, 1181.02)	1905.20 (1854.79, 1942.59)	1393.90 (1291.39, 1501.59)	7017.00 (5883.20, 8111.69)	11098.17 (9239.87, 12841.99)	23002.59 (20122.67, 27166.18)	27463.75 (22839.24, 32676.71)	318.22 (284.55, 347.17)	513.34 (460.16, 559.19)	126.19 (100.86, 158.31)	206.49 (160.59, 263.01)	740.34 (493.74, 975.35)	516.11 (339.53, 692.52)	7.65 (6.02, 9.66)	18.96 (14.93, 23.11)	176.13 (158.17, 198.01)	186.75 (171.07, 203.51)	743.28 (583.36, 979.61)	568.47 (458.48, 688.99)
Percentage of global, %	100.00	100.00	2.23	1.79	11.53	5.87	6.56	9.50	21.84	33.30	0.39	0.58	0.22	0.31	1.45	1.80	0.02	0.04	0.91	0.97	0.25	0.28
Percent change of incidence 1992–2021, %	23.09		−16.06		−26.84		58.16		19.39		61.32		63.63		−30.24		147.84		6.03		−23.52	
All-age incidence rate																						
Rate per 100,000^*^	1174.28 (1102.39, 1251.31)	1006.89 (921.88, 1083.21)	882.04 (860.66, 904.07)	515.10 (490.97, 535.96)	1255.51 (1222.29, 1280.15)	962.28 (891.51, 1036.62)	792.62 (664.55, 916.27)	784.60 (653.23, 907.89)	1906.97 (1668.21, 2252.14)	1930.33 (1605.29, 2296.74)	822.94 (735.87, 897.82)	902.91 (809.36, 983.55)	739.55 (591.10, 927.80)	547.66 (425.92, 697.55)	1287.35 (858.54, 1696.00)	488.61 (321.44, 655.62)	364.58 (287.07, 460.44)	196.87 (155.03, 239.95)	292.85 (262.99, 329.23)	218.79 (200.43, 238.43)	1354.36 (1062.97, 1784.99)	521.83 (420.86, 632.46)
Percent change of rate 1992–2021, %	−14.25		−41.60		−23.36		−1.01		1.22		9.06		−25.95		−62.05		−46.00		−25.29		−61.47	
Age-standardized incidence rate																						
Rate per 100,000^*^	1486.32 (1395.42, 1587.72)	923.64 (844.83, 993.18)	1257.59 (1219.80, 1288.98)	446.04 (424.98, 464.27)	1036.09 (1006.68, 1057.48)	628.62 (582.10, 677.06)	1279.86 (1067.28, 1479.99)	888.62 (733.24, 1030.81)	2708.04 (2369.33, 3177.79)	1351.55 (1129.11, 1600.86)	1234.18 (1071.41, 1361.74)	1050.16 (942.10, 1143.69)	1443.33 (1138.63, 1790.56)	736.06 (597.14, 890.62)	1879.32 (1218.48, 2435.19)	735.75 (477.34, 992.07)	919.94 (722.75, 1165.94)	448.56 (370.04, 536.14)	506.55 (454.59, 554.04)	230.56 (210.89, 251.65)	3076.37 (2445.57, 3923.30)	1186.10 (956.81, 1451.49)
Percent change of rate 1992–2021, %	−37.86		−64.53		−39.33		−30.57		−50.09		−14.91		−49.00		−60.85		−51.24		−54.48		−61.44	
APC model estimates																						
Net drift of incidence rate, % per year^#^	−1.87 (−1.93, −1.80)		−3.73 (−3.83, −3.63)		−2.80 (−3.19, −2.42)		−1.61 (−1.81, −1.40)		−2.57 (−2.74, −2.41)		−1.76 (−2.20, −1.32)		−2.37 (−2.54, −2.21)		−3.35 (−3.58, −3.11)		−2.43 (−3.09, −1.77)		−2.38 (−2.52, −2.23)		−3.99 (−4.07, −3.91)	

All-age incidence rate: crude incidence rate.

Age-standardized incidence rate is computed by direct standardization with global standard population in the GBD 2021.

Net drifts are estimates derived from the age-period-cohort model and denotes overall annual percentage change in incidence rate, which captures the contribution of the effects from calendar time and successive birth cohorts.

*Parentheses for all GBD health estimate indicate 95% uncertainty intervals due to the inherent characteristics of model selection, parameter estimation, and the quality and availability of data inputs for GBD 2021.

#Parentheses for net drift indicate 95% confidence intervals.

APC, age-period-cohort, UI, uncertainty interval, CI, confidential interval.

**Figure 2:**
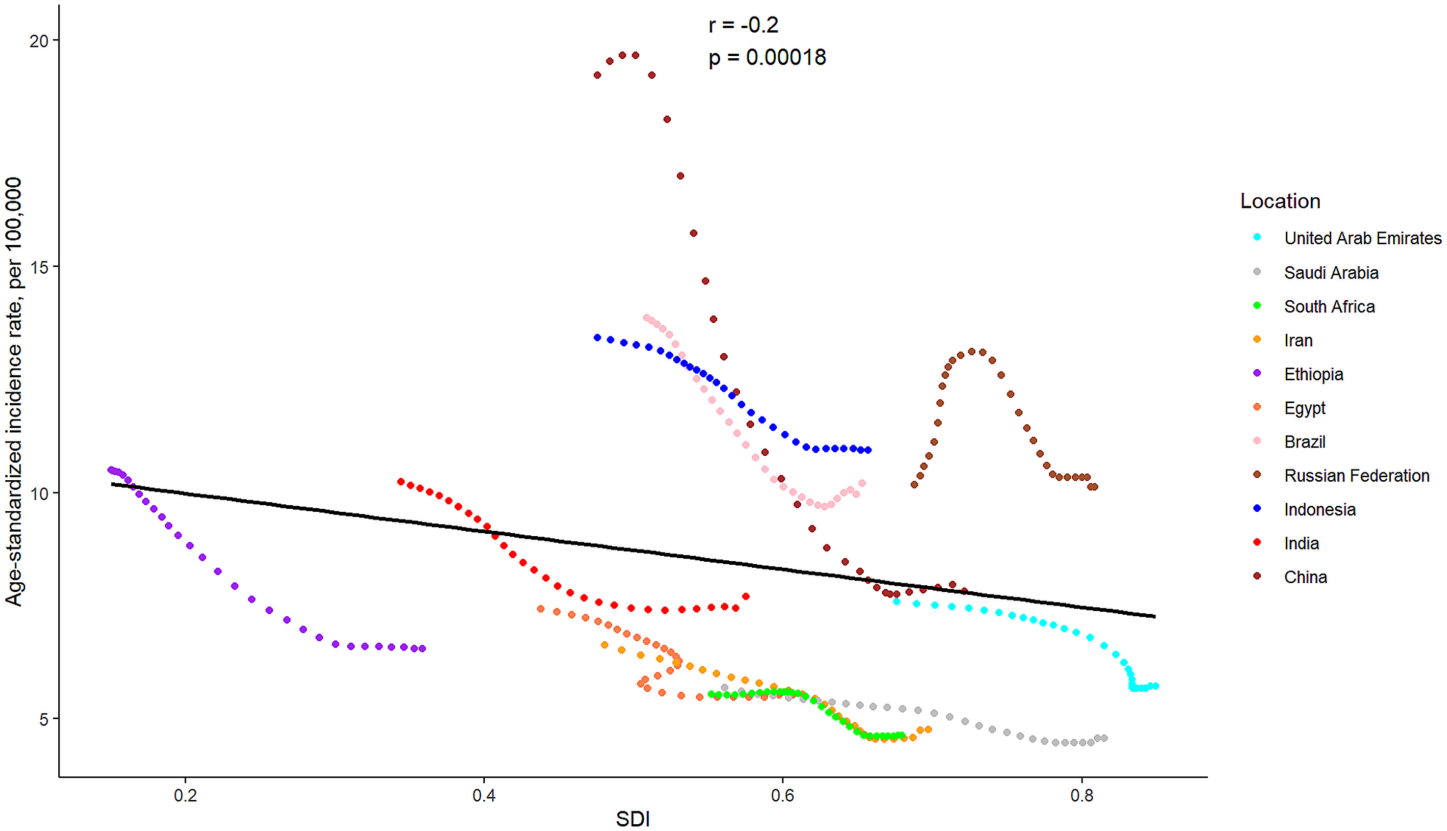
The associations between the SDI and age-standardized incidence rate per 100,000 population of subarachnoid hemorrhage across eleven BRICS countries. SDI, Sociodemographic Index.

Except for China, SAH cases were observed to have significantly increased in the other ten BRICS member countries over the study period. Among these, Indonesia exhibited the most substantial rise, with a 61.28% increase in incidences. In 2021, the all-age incidence rate and age-standardized incidence rate for SAH varied significantly across countries, ranging from 4.25 (95% UI: 3.49 to 5.02) and 4.57 (95% UI: 3.96 to 5.17) per 100,000 population in Saudi Arabia to 10.53 (95% UI: 8.98 to 12.24) and 10.94 (95% UI: 9.54 to 12.64) per 100,000 population in Indonesia, respectively. All BRICS-plus countries demonstrated a declining trend in SAH incidence from 1992 to 2021. Notably, China exhibited the largest decrease in age-standardized incidence rate, with a drop of 59.36%, while the Russian Federation showed the least significant decrease, at 0.52%. According to the APC model estimates, the annual net drift in the SAH incidence rate ranged from −3.36% (95% UI -3.43 to −3.28) for China to −0.50% (95% UI -0.58 to −0.41) for the Russian Federation within eleven countries (Table 1). Furthermore, a significant negative correlation was identified between the age-standardized rate of SAH incidence and SDI values (*r* = −0.2, *p* < 0.001) (Figure 2).

### 3.2 Time trends in SAH incidence across different age groups

Supplementary Figure S1 illustrates the annual percentage change in the incidence rate of SAH across 5-year age groups, ranging from 0 to 94 years. Generally speaking, all age groups exhibited negative local drift values, indicating a global decline in SAH incidence rate. Similar downward trends were also evident in almost all age groups among eleven BRICS members. However, notable exceptions were identified in Brazil and the Russian Federation, where older age groups exhibited pronounced positive local drift values. It should also be noted that females have more age groups (especially the older people) associated with positive net drift values than males in the Russian Federation.

Supplementary Figure S2 depicts the temporal patterns in the number of SAH cases across different age groups. Globally, the majority of SAH cases were predominantly observed among individuals aged 40 years and older, with similar distributions noted across the original five BRICS nations. In contrast, the new BRICS member countries exhibited a broader distribution of SAH cases spanning all age categories. Notably, the age-related distribution of SAH incidences demonstrated relative stability both globally and within most BRICS-plus countries over the period from 1992 to 2021. However, a distinct trend has emerged in Saudi Arabia in recent years, with the burden of SAH shifting from younger adults (20–39 years) to middle-aged individuals (40–64 years).

### 3.3 Age, period and cohort effects on SAH incidence

**Figure 3:**
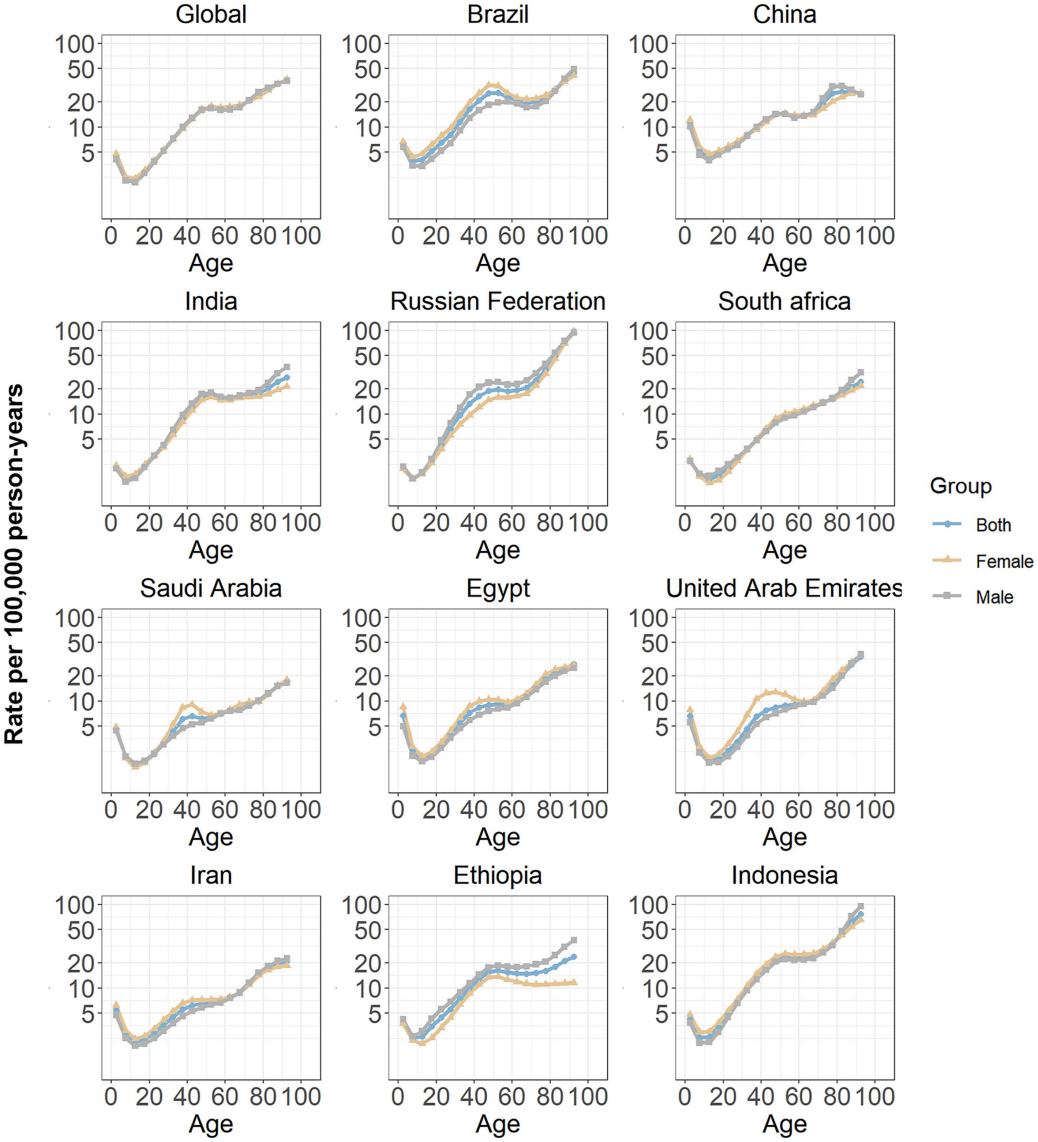
Age effects on subarachnoid hemorrhage incidence in global and BRICS-plus. Age effects are shown by the fitted longitudinal age curves of incidence rate (per 100,000 person-years) adjusted for period deviations. The dots denote incidence rate.

The age effects estimated through the APC model for both global and BRICS-plus countries are presented in Figure 3. Generally, a similar age effect pattern was identified among all analyzed members, and the incidence rate of SAH showed downward and then upward trends with age in the reference cohort after adjusting for period effects, similar to a “V-shaped” curve. Among both males and females, the 90–94 years age group demonstrated the highest incidence rate of SAH.

**Figure 4:**
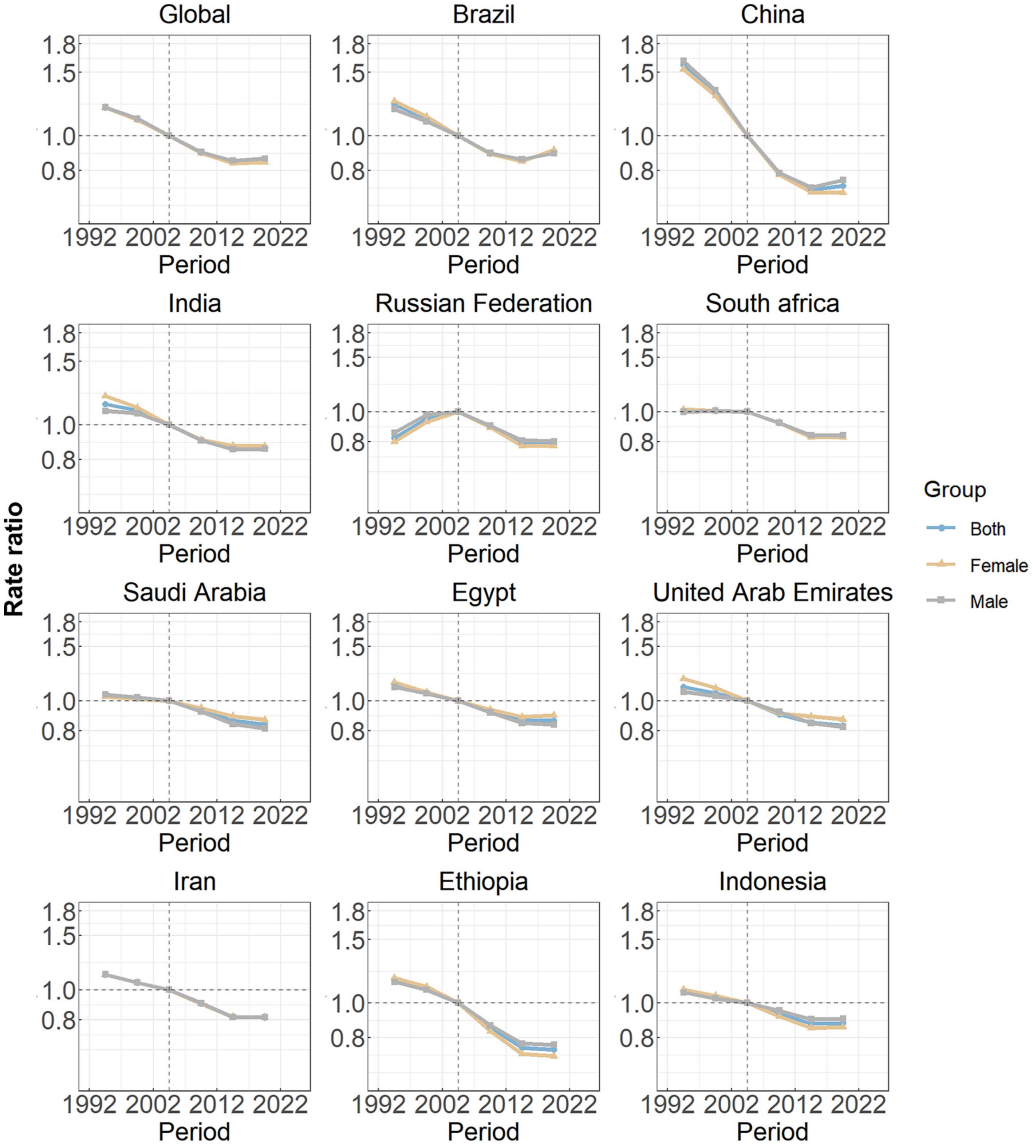
Period effects on subarachnoid hemorrhage incidence in global and eleven BRICS countries. Period effects are shown by the relative risk of incidence rate (incidence rate ratio) and computed as the ratio of age-specific rates from 1992–1996 to 2017–2021, with the referent cohort set at 2002–2006. The dots denote incidence rate ratios.

Figure 4 illustrates the estimated period effects stratified by sex over the study duration. Overall, the period effects exhibited a steady decline, suggesting a continuous improvement in the management of the SAH burden over time. A similar trend was observed in the ten BRICS-plus nations, including Brazil, China, India, South Africa, Saudi Arabia, Egypt, the United Arab Emirates, Iran, Ethiopia, and Indonesia. Among these countries, China demonstrated the most significant reduction in period effects. In contrast, the Russian Federation displayed a distinct pattern, with elevated period risks prior to the reference period (2002–2006), followed by a subsequent decline.

**Figure 5:**
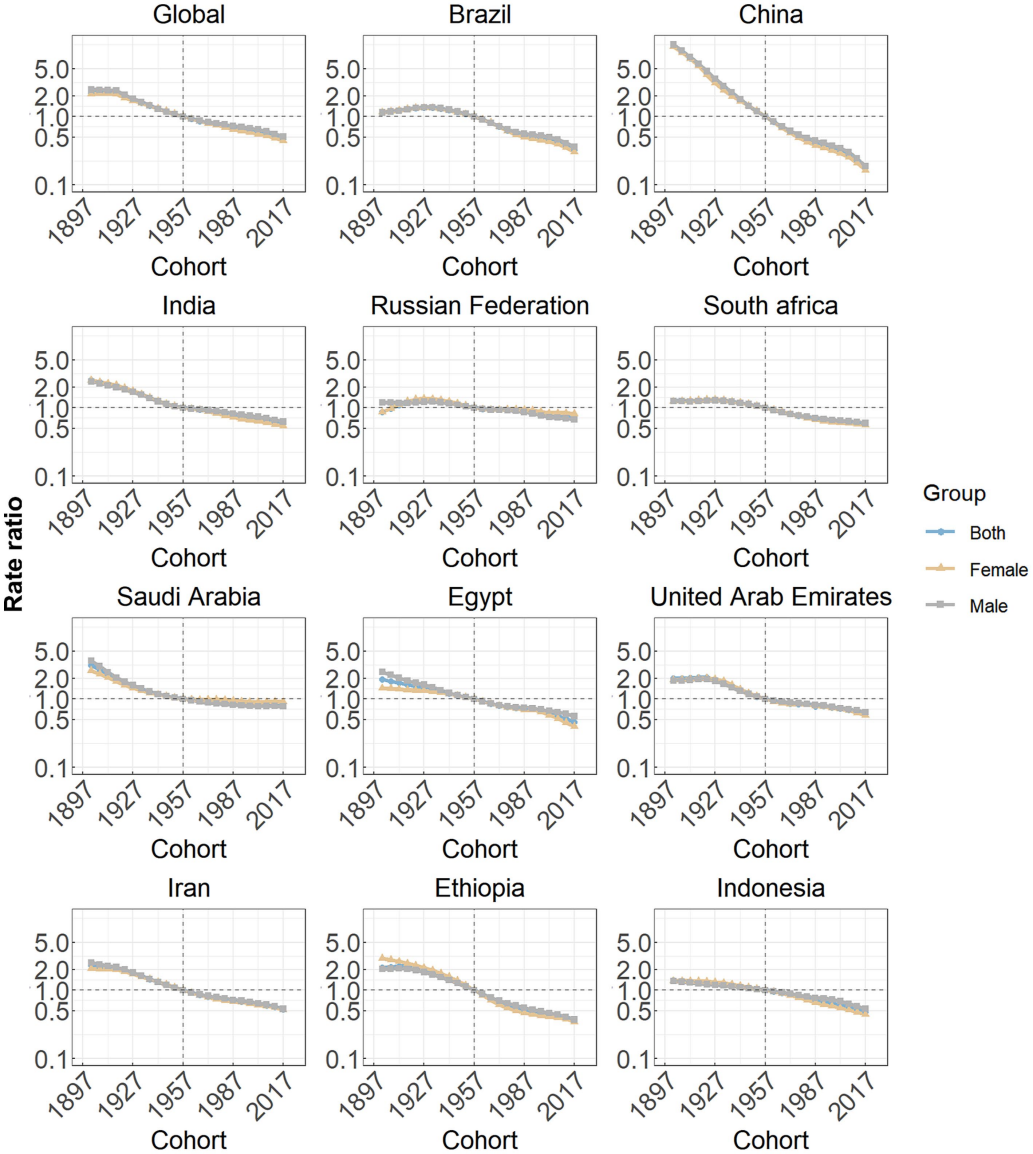
Cohort effects on subarachnoid hemorrhage incidence in global and eleven BRICS countries. Cohort effects are shown by the relative risk of incidence rate and computed as the ratio of age-specific rates from the 1940 cohort to the 2004 cohort, with the referent cohort set at 1972. The dots denote incidence rate ratios.

Cohort effects globally exhibited a continuous decline across successive birth cohorts over the past thirty years, as seen in Figure 5. This pattern was particularly prominent in most member countries (China, India, Saudi Arabia, Egypt, the United Arab Emirates, Iran, Ethiopia and Indonesia). In the Russian Federation, South Africa and Saudi Arabia, the RRs associated with certain birth cohorts initially remained relatively stable and subsequently began a decline.

## 4 Discussion

This investigation highlights significant variations in the global burden of SAH from 1992 to 2021, with a particular focus on the BRICS-plus nations. The results demonstrate a 29.54% rise in the absolute number of SAH cases, contrasting with a 30.05% decline in the age-standardized incidence rate. Notably, considerable heterogeneity was observed among the eleven BRICS-plus member states. These findings further underscore the complex epidemiological patterns of SAH and highlight significant disparities in health outcomes, suggesting potential strategies for prioritizing interventions aimed at reducing the burden of SAH across different age groups, time periods, and birth cohorts within the BRICS-plus framework.

The rise in subarachnoid hemorrhage (SAH) cases over the last 30 years is likely attributable to global population growth. However, significant disparities in SAH incidence rates and long-term trends are evident across BRICS-plus nations. Eight of eleven members show varying degrees of increase in all-age incidence rate, while China, Egypt, and Ethiopia show a decrease. When age distribution is accounted for, the age-standardized incidence rates of SAH among BRICS countries from 1992 to 2021 suggest that factors beyond demographic changes, such as advancements in neurological health programs and progress in medical and public health sectors, have played a critical role in shaping these trends (19, 20). Another aspect is that economic support is often considered a key determinant of healthcare capacity in cerebrovascular disease management (8), and our results may substantiate the rationale for prioritizing interventions based on socioeconomic contexts. The significant negative correlation between SAH burden and SDI highlights the complex influence of early diagnosis, preventive strategies, lifestyle modifications, and enhanced healthcare systems driven by socio-demographic progress. Moving forward, addressing socioeconomic inequities must be central to policies and research aimed at reducing the global burden of stroke more equitably.

The patterns observed in the burden of SAH across different age groups appear to correlate with global improvements in healthcare accessibility and advancements in stroke management protocols. The overall reduction in incidence rates points to the success of strengthened primary prevention measures, enhanced acute care interventions, and more effective rehabilitation programs introduced in recent decades. Similar trends have been observed among BRICS-plus nations, suggesting parallel progress driven by evolving health policies and systemic improvements within these rapidly developing regions. However, in Brazil and the Russian Federation, individuals aged over 80 years demonstrated positive local drift values. This may indicate emerging challenges associated with an aging population and the increasing prevalence of age-related comorbidities (21). Additionally, gender-specific differences were highlighted, with females in the Russian Federation showing more positive net drift values. This warrants further exploration into gender-related risk factors, health-seeking behaviors, and underlying biological mechanisms. Notably, prior studies have suggested that postmenopausal women may face an elevated risk of SAH, potentially due to hormonal changes that negatively impact vascular health (22, 23). Furthermore, societal and healthcare system factors that uniquely influence women’s health may play a role in shaping this observed pattern (23). Our evaluation of age-related trends in SAH cases reveals a concentration within the middle and older age groups (40 years and over), aligning with existing knowledge related to the progressive nature of vascular risk factors such as hypertension (24). In contrast, the recent and notable shift of the SAH cases in Saudi Arabia from older individuals to younger and middle-aged populations raises distinct concerns. This epidemiological change suggests that middle-aged groups may now be increasingly impacted by risk factors that were traditionally more common among younger individuals. Previous studies highlighted significant shifts in lifestyle factors, such as rising rates of obesity, diabetes, and hypertension, within middle-aged populations in the region (25–27). Implementing robust national strategies to target these modifiable risk factors may help address this emerging trend and reduce the disease burden in the future.

Significant disparities in the incidence rates of SAH are observed across different BRICS members. To facilitate informed decision-making, policymakers should assess the unique characteristics of their nations while considering their relative standings in a global context. The triggers for SAH often raise questions, warranting further investigation into age, period, and cohort effects. This study has focused on analyzing the patterns of SAH burden both globally and within the BRICS-plus countries using the APC framework. Elderly individuals experience the most pronounced health impacts related to SAH, with the risk escalating as age advances. Biologically, aging is a well-documented risk factor for intracranial aneurysm formation, growth, and rupture (28). Age-related vascular changes, including endothelial dysfunction and increased arterial stiffness, contribute to the weakening of the arterial wall and heightened susceptibility to rupture (29, 30). Additionally, the cumulative effects of modifiable risk factors, like hypertension and smoking, over a lifetime may further increase the risk among older adults (31). Another important consideration is the global rise in life expectancy, leading to a growing elderly population (12). This demographic transition highlights a shift in prevailing health challenges from infectious diseases to chronic non-communicable conditions, including cerebrovascular disorders.

The decline in period effects observed in most BRICS countries has been attributed to advancements in healthcare infrastructure, heightened public health awareness, and the implementation of targeted interventions that have collectively reduced the SAH burden over recent decades. In China, the pronounced reduction in period effects has been linked to comprehensive healthcare reforms that substantially improved medical care accessibility and quality (32). Policies including the New Rural Cooperative Medical Scheme and Urban Resident Basic Health Insurance have also been instrumental in mitigating the SAH burden (33, 34). Additionally, public health campaigns focusing on controlling hypertension (35) and reducing smoking (36), major risk factors for SAH, are vital elements that have contributed to this progress. India and Brazil have also demonstrated reductions in SAH period effects, though varying in magnitude. In India, the expansion of primary healthcare networks and elevated healthcare investments have underpinned these trends, supported by campaigns addressing non-communicable diseases and gradual socioeconomic improvements (37). Brazil’s progress has been driven by the increased awareness on the risk factors of cerebrovascular diseases among the general public and prioritization of acute cerebrovascular care, leading to declines in SAH incidence and mortality (38, 39). South Africa exhibits a heterogeneous profile, with socio-economic disparities continuing to hinder healthcare accessibility and quality. Recent initiatives aiming to strengthen healthcare systems and increased awareness around hypertension and smoking cessation have contributed to initial declines in SAH risk (40–42). Persistent apartheid-era inequalities, however, remain a barrier to achieving equitable healthcare outcomes nationwide (43).

In Middle Eastern countries including Saudi Arabia and the United Arab Emirates, cultural transformations and economic development have contributed to significant healthcare advancements, particularly in reducing SAH incidence rate. Launched as a health sector transformation initiative, Saudi Arabia’s Vision 2030 prioritizes early prevention strategies, implementation of advanced medical technologies, and enhancement of public awareness regarding SAH-associated risk factors (44). The United Arab Emirates’ substantial investments in healthcare development and preventative medicine programs have demonstrated effectiveness in SAH risk management (45, 46). Comparable trends identified in Egypt and Iran may be explained by recently implemented public health initiatives that emphasize cerebrovascular health improvement and expansion of healthcare accessibility (47, 48). Conversely, the Russian Federation initially demonstrated elevated SAH risks, stemming from historical healthcare system limitations coupled with prevalent lifestyle-related factors such as tobacco use and excessive alcohol consumption (49). However, following the reference period, coordinated tobacco reduction campaigns and healthcare system optimization may have driven subsequent risk reduction (50, 51). These outcomes appear substantially influenced by government-sponsored programs targeting non-communicable disease prevention through health behavior modification initiatives. In Ethiopia and Indonesia, despite persistent challenges in healthcare resource allocation and infrastructure development, the decline in cohort effects highlights progress in minor healthcare interventions and education geared toward reducing SAH risk factors (52, 53). Both nations have demonstrated progressive reorientation of public health priorities, evidenced by increasing emphasis on non-communicable disease prevention (54, 55).

Compared with the recent GBD 2021 reports (21, 56, 57), this study conducts a detailed examination of the global burden of SAH by incorporating age, period, and cohort effects. A key innovation lies in accurately quantifying shifts in age-related burdens over the span of 1992 to 2021, with a focus on both global data and trends specific to eleven BRICS members. This methodology enables an in-depth exploration of incidence patterns within distinct age groups while accounting for influences tied to specific time periods and birth cohorts. However, several limitations should be noted. First, the data for the GBD 2021 were obtained from a variety of sources, including surveys, registries, and administrative records, which exhibited variations in quality and completeness. This heterogeneity may introduce potential biases and uncertainties into the estimates. Second, the GBD database frequently employs modeled estimates for regions with limited empirical data. The assumptions underlying these models may not hold universal validity, particularly when considering the impact of diverse cultural, genetic, and environmental factors on the burden of SAH. Nevertheless, the GBD 2021 remains committed to addressing potential biases in modeling data: (1) a systematic review of the literature was conducted for GBD 2021 which resulted in the addition of 48 new sources for SAH; (2) adjustments for alternative study methods and case definitions were applied to data prior to analysis in DisMod-MR. (3) the severity distribution of stroke and stroke subtypes, and the method for adjusting cross-sectional population-level surveys were added and updated. Third, the application of age–period–cohort analysis raises the possibility of ecological fallacies. To address these limitations, several scientifically plausible hypotheses regarding the causal relationships underlying the temporal patterns of SAH incidence have been proposed, with support from available data and existing evidence.

## 5 Conclusion

This investigation provides a comprehensive analysis of the SAH burden within the BRICS-plus members from 1992 to 2021. It demonstrates the overall decline in age standardized incidence rate, while highlighting the persistent health inequalities among eleven countries that may be driven by socio-economic differences. Furthermore, the findings elucidate the intricate dynamics of age, period, and cohort influences on SAH incidence. Distinct national trajectories, shaped by varying socioeconomic, cultural, and historical frameworks, suggest that public health initiatives must be meticulously customized to mitigate and control the SAH burden across diverse settings.

## Data Availability

Publicly available datasets were analyzed in this study. This data can be found here: the datasets generated during and/or analyzed during the current study are available in the GBD Data Tool repository (http://ghdx.healthdata.org/gbd-results-tool). This public link to the database of GBD study is open, and the use of data does not require additional consent from IHME.

## Footnotes

^1^ https://ghdx.healthdata.org/gbd-2021
