# Supplementary material for: Epidemiological trends and age-period-cohort effects on subarachnoid hemorrhage burden across the BRICS-plus from 1992 to 2021
Source: Front Med (Lausanne). 2025 Jul 3;12:1582357. doi: 10.3389/fmed.2025.1582357 (PMC12267264; doi:10.3389/fmed.2025.1582357)
Supplement: Supplementary file 1 [file Data_Sheet_1.docx]

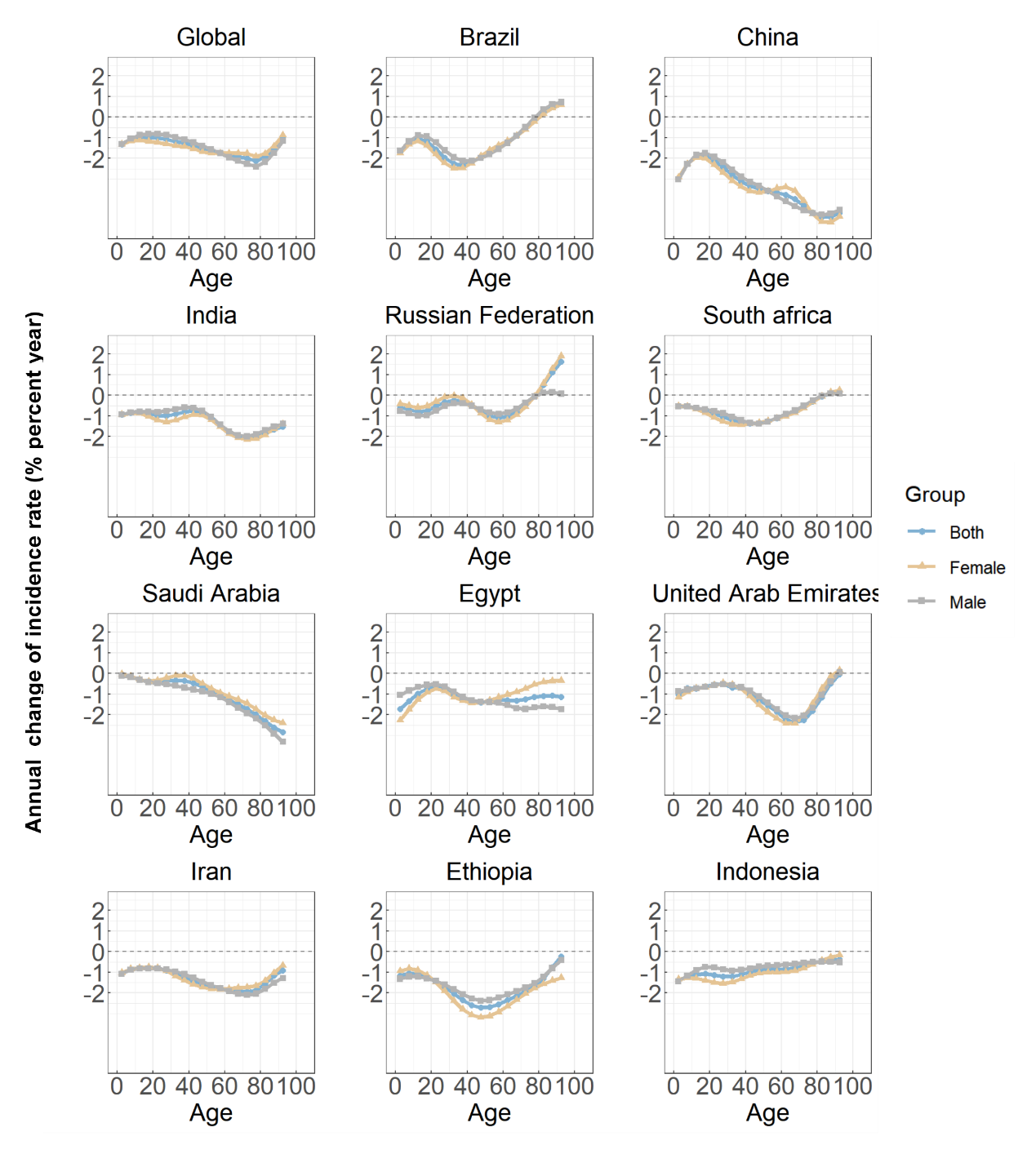


**Figure S1** Local drifts of subarachnoid hemorrhage incidence rate (estimates from age-period-cohort models) for 19 age groups (0-4 to 90-94 years) in global and BRICS plus, 1992–2021.

The dots indicate the annual percentage change of incidence rate (% per year).


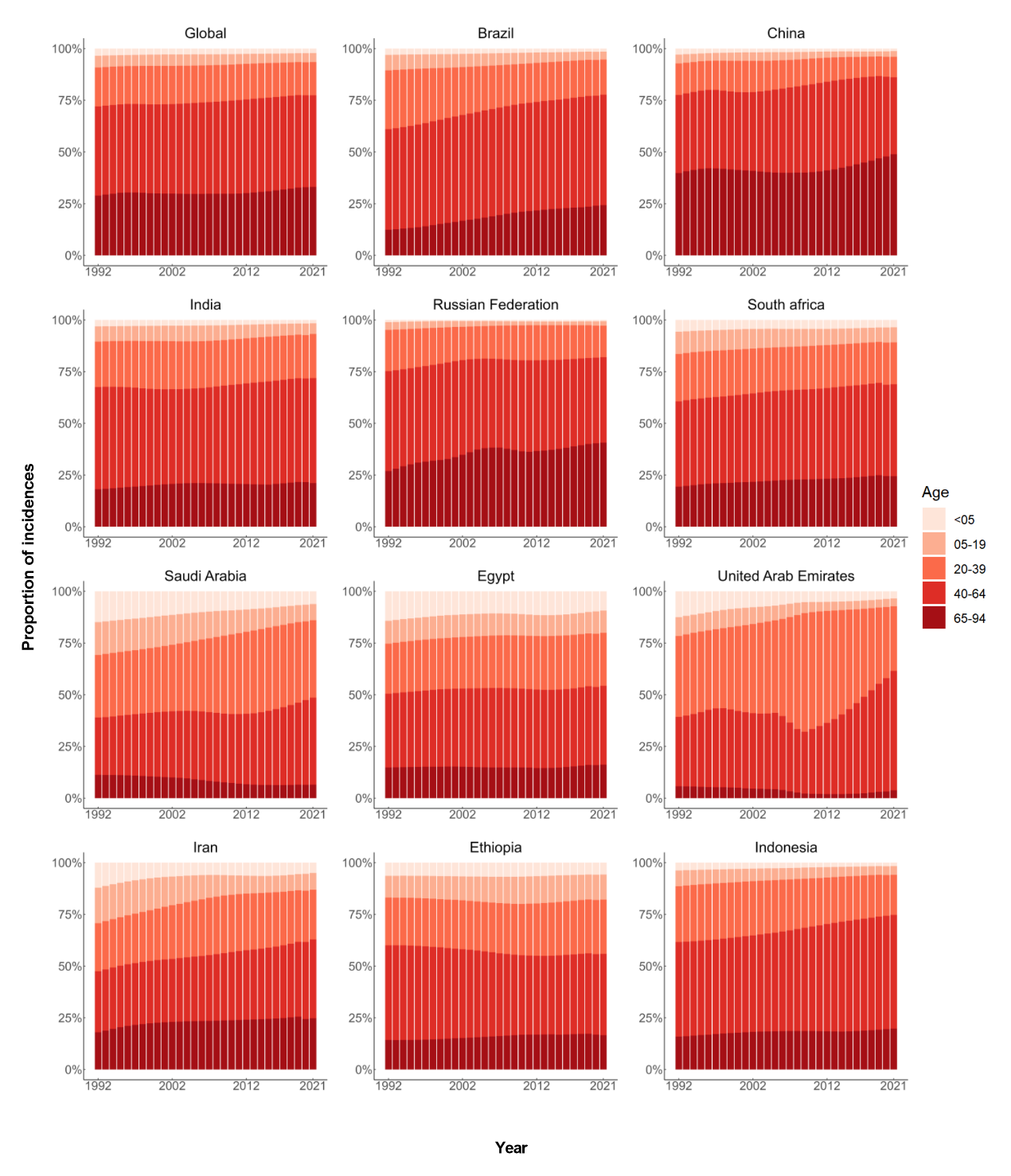


**Figure S2** Age distribution of incidences of subarachnoid hemorrhage in global and BRICS plus, 1992–2021.
